# Reducing stress and anxiety in patients with myocardial infarction with non-obstructive coronary arteries or Takotsubo syndrome: A non-randomized feasibility study

**DOI:** 10.1016/j.invent.2022.100562

**Published:** 2022-07-21

**Authors:** Elisabet Rondung, Sophia Monica Humphries, Erik Martin Gustaf Olsson, Runa Sundelin, Fredrika Norlund, Claes Held, Jonas Spaak, Per Tornvall, Patrik Lyngå

**Affiliations:** a Department of Psychology and Social Work, Mid Sweden University, 831 25 Östersund, Sweden; b Department of Women's and Children's Health, Uppsala University, 751 85 Uppsala, Sweden; c Department of Clinical Science and Education Södersjukhuset, Karolinska Institutet, Stockholm, Sweden; d Department of Medical Sciences, Cardiology, Uppsala Clinical Research Center, Uppsala University, 751 83 Uppsala, Sweden; e Department of Clinical Sciences, Danderyd University Hospital, Karolinska Institutet, Stockholm, Sweden; f Department of Cardiology, Södersjukhuset, Sjukhusbacken 10, 118 83 Stockholm, Sweden

**Keywords:** MINOCA, Takotsubo syndrome, Stress, Anxiety, Cognitive behavioral therapy, Internet-based intervention

## Abstract

**Background and aim:**

In the aftermath of a myocardial infarction with non-obstructive coronary arteries (MINOCA) or Takotsubo syndrome (TS), patients commonly express high levels of stress and anxiety. Current treatment alternatives rarely address these issues. The planned *E-health Treatment of Stress and Anxiety in Stockholm Myocardial Infarction With Non-obstructive Coronaries Study* (e-SMINC) aims to evaluate the effects of an internet-based intervention, building on cognitive behavioral therapy (CBT) by comparison with treatment as usual using an RCT approach. This was a small-scale single arm study designed to test the feasibility of the RCT, addressing uncertainties regarding recruitment, data collection, and intervention delivery.

**Methods:**

Participant recruitment and screening took place before discharge from the coronary care unit at a large Swedish hospital. Eligible patients were invited to a nine-step psychologist guided, internet-based CBT intervention. The sample size was set in advance to 10 participants completing the intervention. The recruitment and flow of participants were documented and evaluated in relation to seven pre-defined progression criteria. Self-reports of anxiety (HADS-A), stress (PSS-14), cardiac anxiety (CAQ), posttraumatic stress (IES-6) and quality of life (Rand-36), collected at screening, pre-intervention and post-intervention, were analysed descriptively and by effect sizes (Cohen's *d*). Individual interviews targeting participant experiences were conducted.

**Results:**

Six out of seven progression criteria yielded no concerns. Out of 49 patients with a working diagnosis of MINOCA or TS, 31 were eligible for screening, 26 consented to participate, and 14 were eligible with regard to symptoms of stress and/or anxiety. Eleven completed the pre-assessment and were given access the intervention, and 9 completed the intervention. Only the number of patients screened prior to eligibility assessment was slightly lower than expected, indicating possible concerns. Self-reports of anxiety, stress, cardiac anxiety, posttraumatic stress, and quality of life all indicated symptom reduction from pre- to post-intervention, generally showing large effect sizes (*d* = 0.6–2.6). The general consensus among participants was that the programme was helpful and relevant, and that the personal contact with the psychologist was highly valued. Setting aside time to complete assignments was found critical.

**Conclusion:**

Conducting a full scale RCT was found feasible. Inclusion of more study sites and minor amendments to the protocol and intervention were decided to improve feasibility further.

**Trial registration:**

Clinicaltrials.govNCT04178434

## Highlights

• Many patients report stress and/or anxiety after a MINOCA or Takotsubo event.
• These issues are seldom addressed in current clinical practice.
• An internet-delivered psychological intervention may be a supplementary tool.
• The piloted procedures for recruitment and data-collection were found feasible.
• The internet-based intervention was found feasible and appreciated by participants.

## 1 Introduction

Myocardial infarction with non-obstructive coronary arteries (MINOCA) presents in patients without accompanying significant coronary artery disease (CAD) and encompasses a subset of patients (6 % according to Collste et al., 2013) with myocardial infarction (MI). Likewise, Takotsubo syndrome (TS) is also presented in cases without significant CAD and although sometimes misclassified under the term MINOCA, TS has a somewhat different physiology and diagnosis. Shared characteristics of patients with these diagnoses compared to MI with CAD often include younger age, female sex, better prognosis, and higher prevalence of previous psychiatric illness (Budnik et al., 2018; Nayeri et al., 2018).

As explored in qualitative interviews (Wallström et al., 2016), stress and anxiety seem to not only be a precursor to MINOCA and TS, but also experienced as a consequence of the event. Despite good prognosis, many patients with TS are still on part-time sick leave after 6 months (Sundelin et al., 2020) and compared to MI with CAD, experience more psychological distress and depressed mood 1 year after the event (Compare et al., 2014). Patients with MINOCA or TS have also reported lower health related quality of life in the domains of vitality and mental health, measured by SF-36, compared with CAD patients (Daniel et al., 2017). The current practice in Sweden after suspected MINOCA or TS does not differ from the follow-up cardiac rehabilitation program offered to patients with MI. This treatment usually includes cardiac rehabilitation focusing on lifestyle changes and physiotherapy, but is not always offered, or not entirely relevant, to patients with MINOCA or TS (Eggers et al., 2018). Taking this into account as well as the high stress and anxiety experienced by this patient group after these events, there is a well-supported unmet need for available psychological interventions following both MINOCA and TS.

Cognitive behavioral therapy (CBT) delivered using web-based intervention programs has been shown effective in reducing both stress and clinical anxiety in adults (Andrews et al., 2018; Heber et al., 2017). There are also indications of small to moderate effects on anxiety and depression in patients with chronic health conditions (McCombie et al., 2015; Mehta et al., 2019) and a few studies evaluating internet-based CBT programs specifically for patients with MI (e.g. Johansson et al., 2019; Norlund et al., 2018). Hence, we were interested in exploring if an internet-delivered CBT-intervention could be effective in reducing stress and anxiety also in patients with MINOCA or TS. The intervention was developed in collaboration with patient research partners (PRPs) all of whom had a previous diagnosis of MINOCA or TS. Building on PRP reports and literature findings, it was designed to target issues of stress and anxiety. Content and functionality of the intervention was tested by the PRPs who provided feedback and suggestions for improvements. A version of the intervention and the process of its design and conception was completed ahead of testing for feasibility and is described elsewhere (Humphries et al., 2020). The collaboration with PRPs is stressed by the UK Medical Research Council's (MRC) framework for development of complex interventions (Craig et al., 2013), in which they highlight the importance of careful preparations before going to a potentially expensive and demanding fully-powered evaluation trial to avoid research waste (Moher et al., 2016). In line with these recommendations, proper feasibility evaluations are important.

### 1.1 Objective

The aim of this study was to evaluate the feasibility of conducting a randomized controlled trial (RCT), comparing the effects of treatment as usual (TAU) with TAU plus an internet-based CBT intervention. For patients with high levels of stress and/or anxiety in the aftermath of a MINOCA or TS event. Using both quantitative and qualitative approaches, we specifically sought to address uncertainties relating to the feasibility of recruitment and data collection, along with feasibility and experiences of the intervention (intervention adherence, psychologists' activities, and participant's views and evaluations). See Table 1 for a full list of the specific research questions addressed. While this study was neither designed nor powered to evaluate any intervention effects, we still explored the appropriateness of the outcome measures by describing changes in self-report data between all data collection points as part of our analysis of the feasibility of data collection.

**Table 1:**
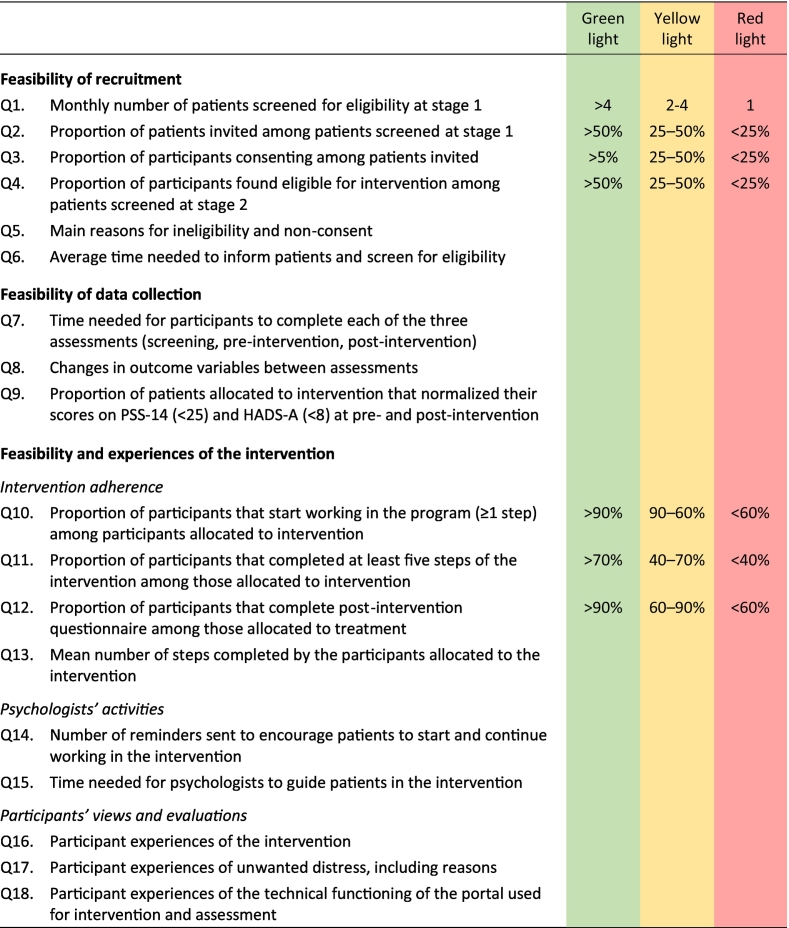
Specific research questions and pre-specified progression criteria of the current feasibility study.

Note. Green light: No concerns regarding feasibility and/or acceptance, no need for further analysis or amendments. Yellow light: Possible concerns relating to feasibility and/or acceptance, need for further analysis, minor amendments might be necessary. Red light: Serious indication of low feasibility and/or acceptance, need for further analysis. Major amendments or discontinuation of trial might be necessary.

The progression criteria were set to be analysed separately rather than in conjunction.

PSS-14 = Perceived Stress Scale, 14-item version; HADS-A = Hospital Anxiety and Depression Scale, Anxiety subscale.

## 2 Methods

### 2.1 Trial design

This was a single arm external feasibility study of the planned e-SMINC randomized controlled trial, registered at Clinicaltrials.gov (NCT04178434) November 26, 2019. Ethical approval was first obtained from the Swedish Ethical Review Authority (Dnr: 2018/1434-31) on August 22, 2018. An amendment was approved March 25, 2020 (Dnr: 2020-00818). With the exception of randomisation, the study followed the protocol outlined for the planned RCT trial at the time. The final study protocol was modified based on the results of this feasibility study and an amendment was approved October 26, 2021 (Dnr: 2021-05351-02).

### 2.2 Progression criteria

In line with recommendations in the Consolidated Standards of Reporting Trials (CONSORT) extension to randomized pilot and feasibility trials (Eldridge et al., 2016), we developed a traffic light model of predetermined progression criteria to guide the decision of whether to proceed, to proceed with amendments, or not to proceed to a full-scale randomized trial (Avery et al., 2017). These progression criteria were set prospectively by a consensus reached in the study steering committee, taking into account the possibility of finalizing the recruitment of participants in 2 years and having an activity level high enough to draw conclusions about the use of the program. The final progression criteria, along with all other specific research questions are presented in Table 1. In addition, study personnel were encouraged to keep note of any unforeseen issues of potential importance to feasibility in a full-scale RCT.

### 2.3 Sample size

The sample size was not set to allow for statistical inferences regarding possible intervention effects, but rather to give opportunity to test study procedures and collect qualitative data of participant experiences of using the intervention. With these aims in mind, we believed that having ten participants using the intervention program would be sufficient. That would also allow us to estimate the number needed to approach in order to recruit the target number of 80 evaluable participants for the full trial.

### 2.4 Setting

The study recruitment and screening took place at Södersjukhuset, one of the largest hospitals in Stockholm, Sweden.

All questionnaires were administered using the Uppsala University Psychosocial Care Program (U-CARE) Portal (the portal), which was also used for intervention implementation. The design of the portal allows for digital data collection and remote delivery of e-Health interventions. It requires double-authentication or a digital secure (BankID) authentication and ensures user anonymity through the use of unique ID numbers that can only be matched to the personal identification number by a researcher with approved access.

This feasibility study was conducted during the covid-19-pandemic. During this time, The Public Health Agency of Sweden had a number of general recommendations, for example to limit the number of encounters and maintain social distance. Older people (aged 70 and above) and people belonging to an at-risk group (including cardiovascular disease) were advised to avoid social contact as much as possible. As a consequence, non-necessary visits to hospital e.g., visits due to participation in research studies like the present study, were cancelled.

### 2.5 Recruitment and participants

Patients admitted to the coronary unit were screened for eligibility. Patients had to have a verified MI or TS and to have undergone coronary angiography with <50 % stenosis within 1 month of the acute event. To be eligible, they further had to be between 30 and 80 years old, have sinus rhythm on ECG at admission, and to score ≥ 8 on HADS-A (Zigmond and Snaith, 1983) and/or ≥ 25 on PSS-14. The PSS cutoff was based on a Swedish norm value (Eskin and Parr, 1996), previously used to distinguish between low and high stress (Brinkborg et al., 2011). Exclusion criteria were acute myocarditis, acute pulmonary embolism, acute MI type 2, previous MI due to CAD, cardiomyopathy other than TS, severe kidney disease and severe pulmonary disease. Not having computer or internet access, or willingness or knowledge to use these, as well as insufficiency in Swedish language were also exclusion criteria. All participants signed a written informed consent before screening at stage 2.

### 2.6 Data collection

#### 2.6.1 Questionnaires

Self-reported demographic characteristics were gathered as part of the screening and collected before discharge from the coronary care unit (CCU). Measurements of trial outcomes were administered pre- and post-intervention, i.e. 2 and 12 weeks after discharge from the CCU respectively. The participant was timely prompted by an e-mail and a telephone text message to complete assessments. After completion of pre-intervention assessments, the participant got immediate access to the intervention program. If non-respondent, reminders were sent 1 week after the original prompt, and if participants still did not respond after one more week, they were contacted by telephone. Screening (stage 2), pre- and post-intervention questionnaires included severity of perceived stress (PSS-14; Cohen et al., 1983), severity of anxiety and depressive symptoms (HADS-A and HADS-D; Zigmond and Snaith, 1983), quality of life (Rand-36; Orwelius et al., 2018), symptoms of cardiac anxiety (CAQ; Eifert et al., 2000), and severity of posttraumatic stress symptoms (IES-6; Thoresen et al., 2010). At post-intervention, participants were also presented with open-ended questions where they could give free-text written feedback on the intervention and study.

#### 2.6.2 Interviews

Individual telephone interviews were conducted with all participants that had worked actively with the intervention program. To encourage more open feedback, the interview was conducted by a psychologist that was not assigned to the participant during treatment. A semi-structured interview guide was used, covering experiences of areas such as the content and material, assignments, reminders, psychologist contact, technical functioning, and potential negative consequences of the treatment. The interviews were recorded on Dictaphone or computer and transcribed verbatim.

#### 2.6.3 Study logs

Data on the flow of participants were logged throughout the study. Reasons for ineligibility or non-consent were documented continuously by a research nurse, along with the time needed for study personnel to inform about the study and assess eligibility. Number of patients completing the different assessments and the time needed to do so were documented automatically in the portal. The psychologists logged the number of intervention steps completed, the number of reminders, and the time used for patient guidance. All personnel working in the trial also made personal notes of any ideas on how the study procedures could be refined before finalizing the RCT protocol.

### 2.7 Intervention

The guided internet-delivered self-help program, developed in close collaboration with PRPs (Humphries et al., 2020), was based on CBT principles and specifically designed to address issues of stress and anxiety among patients with MINOCA and TS. It was implemented using the portal, where the participants could work with the nine steps of the program, access additional material and stay in touch with their psychologist using an internal message system. Participants were given access to the first six steps of the intervention program once they had finalized their pre-intervention assessments. They were recommended to do one step per week.

As summarized in Table 2, the nine steps of the program focused on self-monitoring and identification of stress behaviors and life stressors, recovery and relaxation, personal values, heart-related worry, fear and avoidance (including exposure), and relapse prevention. Steps 7–8 were optional depending on the patient's experience of heart-related worry or fear.

**Table 2:** Overview of the guided and internet-delivered self-help program.

Step	Content and assignments
1	To have had a MINOCA/TS - Introduction to the program - Information about MINOCA/TS and common psychological reactions - Assignment: Describe experiences of having had a MINOCA/TS, formulate treatment goals
2	Stressors and stress behaviors - Common stress reactions and stressors - Positive and negative sides of stress - Assignment: Describe general stress reactions - Assignment: Identify current life stressors
3	Short and long term consequences of actions - Basic principles of positive and negative reinforcement of behaviour - Assignment: Self-monitoring of specific stress situations
4	Recovery and relaxation - General information about recovery, rest, sleep and relaxation - Examples of recovery activities and a relaxation exercise - Assignment: Plan this week's recovery and relaxation activities
5	Personal values - Why values give important guidance in life - Values in work/education, leisure, relationships and personal growth/health - Assignment: Identify values in each domain and plan committed actions in one
6	Fear and avoidance post MINOCA/TS - Common fear reactions following a MINOCA/TS - Generalization of fear, negative effects of avoidance and safety behaviors - Assignment: Describe personal fear situations and possible gains of challenging fear
7	Exposure of cardiac related fear, part 1 (optional) - Principles of fear exposure - Assignment: Formulate an exposure hierarchy. Plan and start exposure training
8	Exposure of cardiac related fear, part 2 (optional) - Assignment: Continued exposure training
9	Conclusion, maintenance and relapse prevention - Assignment: Summarize learnings and further needs. Plan how to maintain gains and continue development. - Relapse prevention - Assignment: Plan for relapse prevention and handling - Guidance on possible ways to additional support

MINOCA = Myocardial infarction with non-obstructive coronary arteries, TS = Takotsubo syndrome.

Each step was built on a combination of informational material (texts and video clips), case examples, and assignments – all in Swedish. All steps included a very brief assessment of symptoms of anxiety and depression (PHQ-4; Kroenke et al., 2009). This scale was only used to inform psychologists of possible need of urgent contact with the participant.

On completion of the first step, the therapists contacted each participant by telephone. The aim of this call was to establish report, define possible treatment goals, and decide what day of the week the participant would submit their assignment reflections. In the remaining steps of the program, the therapists gave their participants personal written feedback on their assignments. Based on the assignments in step 6, the therapist decided whether to recommend steps 7–8, or if the participant could move directly to step 9. Full intervention adherence could thus mean having completed either 7 or 9 steps. If a participant had completed five intervention steps or more, we defined the intervention as completed per protocol.

Reminders were sent manually following a predefined protocol. One to 2 days before an assignment was to be submitted, the therapists sent a brief reminder by a text message. If an assignment was not reported, reminders were sent at one, five, and 8 days. After that, the therapist tried to reach the participant by telephone.

### 2.8 Analytical methods

Quantitative data were primarily analysed using descriptive statistics. To give a hint of changes in outcomes between assessments we calculated within group effect sizes using Cohen's *d* with 95 % confidence intervals. Individual mean imputation was applied in cases where <20 % of a scale's items were missing (Shrive et al., 2006).

For qualitative data, including free-text responses, interview data, and researcher logs, we summarized and coded text to create themes inspired by methods of conventional content analysis (Hsieh and Shannon, 2005).

Analysis of issues encountered either by the progression criteria or in qualitative analyses was inspired by A process for Decision-making after Pilot and feasibility Trials (ADePT; Bugge et al., 2013). Issues were categorised as to whether they were likely to be problematic in the trial only (Type A), in both the trial and a real world setting (Type B), or in a real world setting only (Type C). In a next step, possible solutions were identified and evaluated at research group meetings, guiding amendments based on their potential effectiveness and feasibility.

## 3 Results

### 3.1 Feasibility of recruitment (Q 1–6)

**Fig. 1:**
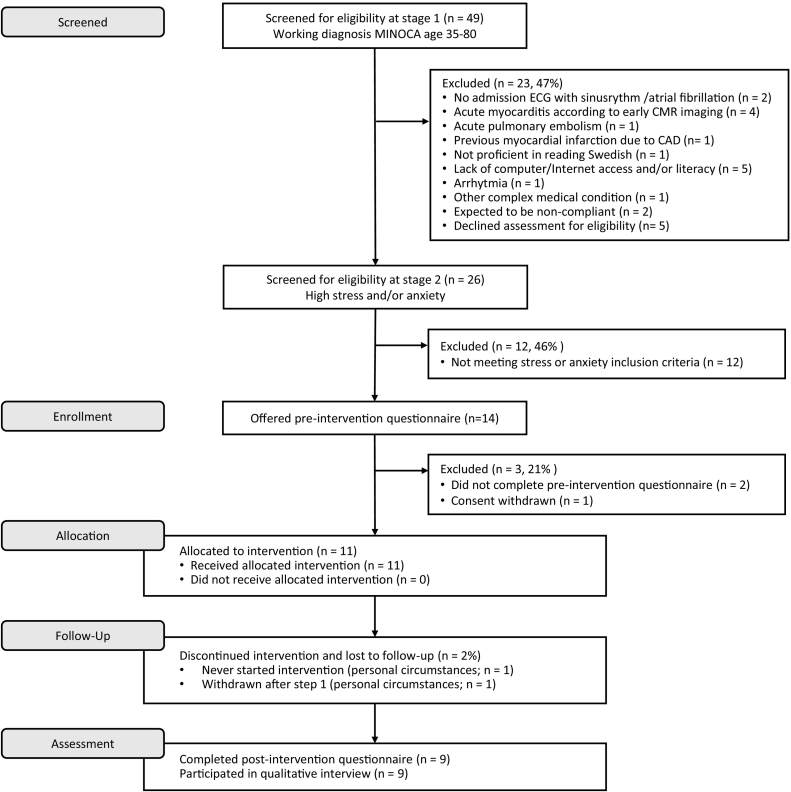
CONSORT Flow diagram showing the flow of participants through the trial.

Recruitment took place between December 9, 2019 and April 13, 2021. On average, hospital personnel needed approximately 90 min to inform about the study and assess eligibility (Q6 in Table 1). Fig. 1 shows the flow of participants throughout the study. During the 16 months of recruitment, 49 patients were screened for possible eligibility at stage 1 (Q1: 49/16). Of these, 18 were excluded due to not fulfilling our inclusion criteria, and of the remaining 31 invited (Q2: 31/49), 26 consented and were screened for eligibility regarding stress and/or anxiety (stage 2; Q3: 26/31). The main reasons for non-consent were lack of time or interest. One patient was moving abroad (Q5; for details on ineligibility see Fig. 1).

Twelve patients reported symptoms below our predefined cut-off point regarding stress and anxiety, and were thus found ineligible. Of 14 eligible patients (Q4: 14/26), two did not complete the pre-intervention questionnaires and one decided to withdraw consent before the intervention started. These three did not have Swedish as their native language. Thus, 11 participants were offered the intervention and included in the study.

#### 3.1.1 Participant characteristics

The included participants (*n* = 11) had a mean age of 64.3 years and were mostly women. Seven had experienced a TS event, and 4 were diagnosed with MINOCA. All were married or living with a partner and most were born in Sweden. Most were employed at the time of the event and the remaining were retired. Two participants reported a known family history of MINOCA/TS and five reported that they were receiving treatment for anxiety, depression or low mood at the time of the event. Two participants reported that they were regularly or irregularly receiving ongoing individual or group psychological therapy from a psychologist or councillor at the time of screening. Further background and sociodemographic details are given in Table 3.

**Table 3:** Descriptive background health and sociodemographic data for the participants included into the study (n = 11).

	*n* (%)
Sex	
Female	9 (81)
Male	2 (18)
Marital status	
Single	0
Married or living with partner	11 (100)
Other	0
Country of birth	
Sweden	9 (81)
Outside Sweden	2 (18)
Education level	
Primary	4 (36)
Secondary	3 (27)
University ≤3 years	2 (18)
University >3 years	2 (18)
Employed at the time of event	
Yes	7 (63)
Retired	4 (36)
Smoking status	
Never smoked	5 (45)
Ex-smoker	6 (54)
Smoker	0
Exercise intensity	
Mostly sedentary	1 (9)
Lightly active	2 (18)
Moderate active	7 (63)
Very active	1 (9)
Family history Takotsubo and/or MINOCA	
Yes	2 (18)
Unsure	4 (36)
No	5 (45)
Family history MI (<65 years)	
Yes	2 (18)
Unsure	1 (9)
No	8 (72)
Receiving treatment for (at time of admission)	
Chronic illness	1 (9)
High blood pressure	4 (40)^a^
High cholesterol	1 (10)^a^
Diabetes	0^a^
Anxiety, depression or low mood	5 (45)
Ongoing psychological therapy (reported during screening)	
No	9 (81)
Yes, irregular or infrequent	1 (9)
Yes, regularly	1 (9)
Experienced distress at time of event	
No	2 (18)
Yes, physical stress	3 (27)
Yes, psychological stress	3 (27)
Yes, physical and psychological	3 (27)

a Percentage refers to the value from the valid responses, *n* = 10. MINOCA = Myocardial infarction with non-obstructive coronary arteries; MI = myocardial infarction.

### 3.2 Feasibility of data collection (Q 7–9)

The participants used a mean time of 43, 17 and 30 min to complete the screening (stage 2), pre-intervention and post-intervention assessments, respectively (Q7). Table 4 shows mean ratings at screening, pre-treatment and post-treatment, and changes of five outcome measures, including their subscales, for the nine participants that completed the intervention and the post-assessment (Q8). Small changes over time were observed between screening and pre-intervention assessments, most which were in the positive direction of change, but some displayed a worsening of symptoms between screening and pre-intervention (e.g. several RAND-indices, HADS-D and CAQ total score). Generally, the Cohen's *d* effect sizes for the changes between screening and pre-intervention were small. Between pre- and post-intervention, reports on all assessed measures changed in a positive direction. Both the mean for the HADS-A and PSS total score were below the mean cut-off for inclusion at post-intervention and revealed large Cohen's *d* effect sizes between pre- and post-intervention (HADS-A: *d* = 1.0, PSS: *d* = 1.5). The proportion of patients allocated to intervention that normalized their scores on PSS-14 (<25) and HADS-A (<8) at post-intervention were 6 out of 8 (1 missing) for PSS (75 %), and 6 out of 9 for HADS-A (67 %) (Q9). Five of the nine participants normalized their scores below the cut-off for inclusion for both the PSS and HADS-A post-intervention, and two participants normalized scores on one of the two outcome measures. Four participants thus scored above the threshold for inclusion on at least one of the scales at post-intervention. None of the participants had normalized their scores on these measures at pre-intervention.

**Table 4:** Changes in five outcome measures assessed at screening, pre-treatment and post-treatment (*n* = 9).

	Screening	Pre-intervention	Post-intervention	Screening to Pre		Pre to Post	
Measure	Mean (SD)	Mean (SD)	Mean (SD)	Diff.	Cohen's *d* (95 % CI)	Diff.	Cohen's *d* (95 % CI)
HADS							
HADS-D	8.0 (1.7)	8.4 (2.3)	3.8 (2.7)	0.4	0.14 (−0.21, 0.79)	−4.7	−1.56 (−2.53, −0.54)
HADS-A	11.7 (2.4)	9.4 (2.9)	4.7 (4.4)	−2.2	−0,63 (−1.34, 0.11)	−4.8	−1.03 (−1.83, −0.19)
PSS-14 total	31.8 (6.1)	30.0 (5.9)	18.5 (3.0)^c^	−1.8	−0,34 (−1.00, 0.35)	−11.6	−1.51 (−2.53, −0.45)

CAQ							
Total	24.4 (9.5)	26.2 (5.5)	10.7 (5.5)	1.8	0.15 (−0.51, 0.80)	−15.6	−2.6 (−3.98, −1.17)
Avoidance	8.3 (4.6)	9.8 (2.9)	4.1 (2.0)	1.4	0.26 (−0,42, 0.91)	−5.7	−1,50 (−2.46, −0.51)
Fear	11.3 (4.8)	10.9 (4.3)	4.0 (4.1)	−0.4	−0.07 (−0.73, 0.58)	−6.9	−1.51 (−2.47, −0.51)
Attention	4.8 (2.3)	5.6 (1.7)	2.6 (1.6)	0.8	0.22 (−0.45, 0.88)	−3.0	−1.55 (−2.52, −0.54)

IES-6							
Total	11.9 (3.1)	10.4 (1.9)	3.9 (2.2)^c^	−1.4	−0.32 (−0.98, 0.36)	−6.8	−2.05 (−3.29, −0.77)
Avoidance	3.2 (1.4)	3.3 (0.7)	1.3 (1.3)^c^	0.1	0.07 (−0.58, 0.72)	−2.0	−1.87 (−3.03, −0.67)
Hyperarousal	4.0 (1.7)	3.2 (1.6)	1.1 (0.9)^c^	−0.8	−0.25 (−0.91, 0.42)	−2.3	−1.00 (−1.84, −0.12)
Intrusive	4.7 (1.8)	3.9 (1.5)	1.5 (1.1)^c^	−0.8	0.32 (−0.98, 0.36)	−2.6	−1.32 (−2.26, −0.33)

Rand-36							
Emotional wellbeing	58.2 (12.8)	61.3 (14.0)	78.0 (14.0)^c^	3.1	0.16 (−0.50, 0.82)	17.0	0.90 (0.05, 1.71)
Energy/fatigue	42.5 (14.8)^a^	38.8 (18.2)^b^	68.8 (14.3)^c^	−3.8	−0.19 (−0.89, 0.52)	28.8	1.71 (0.57, 2.80)
General health	55.6 (15.7)	51.7 (13.7)	67.5 (15.6)^c^	−3.9	−0.26 (−0.92, 0.41)	14.3	1.61 (0.54, 2.65)
Pain	61.0 (28.2)	59.2 (26.3)	85.3 (18.4)^c^	−1.8	0.05 (−0.70, 0.60)	21.3	0.76 (−0.05, 1.54)
Physical function	64.4 (33.9)	50.6 (32.1)	71.7 (28.9)	−13.9	−0.64 (−1.35, 0.10)	21.1	0.93 (0.12, 1.71)
Role emotional	55.2 (33.3)	36.8 (25.0)	85.0 (24.4)	−18.4	−0.63 (−1.33, 0.11)	48.2	1.01 (0.18, 1.81)
Role physical	41.7 (35.4)	11.1 (18.2)	66.7 (37.5)	−30.6	−0.78 (−1.52, −0.01)	55.6	1.20 (0.31, 2.05)
Social functioning	68.1 (25.8)	53.8 (15.5)	77.4 (19.5)^c^	−14.3	−0.44 (−1.12, 0.26)	20.6	0.59 (−0.18, 1.33)

Diff = Difference; CI = Confidence interval; HADS = Hospital Anxiety and Depression Scale; PSS-14 = Perceived Stress Scale 14 item version; CAQ = Cardiac Anxiety Questionnaire; IES-6 = Impact of Event Scale 6 item version.

Change reflects observed values.

a Missing data at screening, n = 1.

b Missing data at pre-intervention, *n* = 1.

c Missing data at post-intervention, *n* = 1.

### 3.3 Feasibility and experiences of the intervention (Q10–18)

#### 3.3.1 Intervention adherence (Q10–13)

Of the eleven participants allocated to the intervention, ten started working in the programme (completed ≥1 step; Q10: 10/11) and one withdrew after finishing the first intervention step. The latter participant referred to pain relating to another medical condition as the reason for withdrawal. The remaining nine participants all completed the pre-defined goal of five steps or more and the post-intervention questionnaire (Q11 and Q12: 9/11). The mean number of steps completed was 6.9 (Q13). Only one participant expressed a need to engage in the optional intervention steps focusing on exposure to reduce heart-related fear. Six participants completed the full intervention (7 or 9 steps, depending on if optional steps were included).

#### 3.3.2 Psychologists' activities (Q14–15)

After inviting the eleven participants to the intervention, the psychologists sent an average of 1.0 (range 0–3) reminders for participants to complete the first intervention step (Q14). Participants then received weekly reminders to complete the following steps, and an additional average of 2.2 (0–5) reminders due to inactivity. On rare occasions (3 in total), the psychologists also reminded participants of unread feedback or messages waiting for them in the portal. Table 5 gives an overview of psychologist activities during the trial, and the approximate time needed to complete these (Q15). For a standard participant completing seven steps of the intervention, the psychologists used approximately 20 min on research administration, 2 h on intervention administration, and 2.5 h on treatment-related activities.

**Table 5:** Overview of psychologist activities and time needed to guide participants through the intervention.

Psychologist activities	Time per occasion and patient (minutes)	Total time^a^ (minutes)
Research administration		
Prompt questionnaires	10 min	2 × 10 = 20

Intervention (administration)		
Send welcome messages	10 min	10
Weekly administration (check patient activity, reminders)	15–30 min	7 × 15 = 105
Guide patient through technical issues	10–20 min	
Additional telephone call (administrative)	10–20 min	

Intervention (treatment)		
Telephone contact after Step 1	30 min	30
Personalized written feedback	15–20 min	6 × 20 = 120
Additional telephone call (treatment)	30 min	

a For standard patients completing Steps 1–6 + 9, in total 285 min (4 h 45 min).

The psychologists noticed that all participants who did not complete the full intervention were lost after step 6, when they needed to activate the last part of the intervention manually. The psychologist also noticed a need for minor amendments in some of the intervention materials (i.e. questions that were difficult for participants to understand).

#### 3.3.3 Participants' views and evaluations (Q16–18)

All of the nine participants who completed the intervention, agreed to provide verbal feedback in the form of a telephone interview. In brief, the general consensus was that the programme was good, helpful, and relevant (Q16). Having control over the assignments, time and place of the programme were particularly appreciated. Stress content was brought up by many as being relevant, as were presenting videos and interviews as examples.

The assignments were generally well received. Most participants acknowledged that setting aside enough time to do assignments was necessary. Some mentioned feeling stressed or overwhelmed by having to work on the program. For one participant, this was expressed as contributing to unwanted distress. No other instances of unwanted distress were reported (Q17).

Contact with the psychologist and feedback given on written exercises were positively regarded from all interviewees. Participants reported feeling encouraged and appreciated receiving feedback and many felt that the personal contact with the psychologist was critical to their positive experience of the program.

In addition to the verbal interviews, participants gave written feedback in the form of open-ended questions on the Portal. These additionally focused on which aspects of the treatment they found most meaningful and any suggested improvements. A summary of these findings are presented together with a summary from the interviews in Table 6. With regard to the technical functionality of the Portal (Q18), the participants found it easy to navigate. Problems were few, and when encountered, they were easily resolved. The interface was mentioned to suit better for a computer rather than a smaller device.

**Table 6:** Summary of feedback given from participants after completing the programme based on telephone interviews and written evaluations (Q16,18).

Interview topic	Positives	Negatives
Format	• Length was deemed to be ideal by nearly all • Control over timing and place were appreciated • Reminders were appreciated • Assignments were received well	• Some needed longer to get started with the programme • Reminders could be perceived as stressful at times
Material and content	• Fictional videos and video interviews with health care personnel and a patient representative that had worked with the program were recognised as being useful, even if not utilised • Stress content viewed as relevant and helpful • Exercises to handle stress were practical • Medical information was appreciated • Text examples helped clarify things	• Library was not utilised by the majority • Written tasks could be perceived as stressful • Concentration required sometimes led to stress
Psychologist contact	• Regarded as extremely important and valuable • Described as very motivational and encouraging • Gave many an incentive to continue and complete assignments • Helped participants to feel valued	• Some would have like more/longer contact
Technical functioning (Q18)	• Easy to navigate; worked well • Problems were uncommon but easily resolved when they occurred	• May best be suited for a computer rather than a smaller device

### 3.4 Progression criteria

Table 7 shows the recruitment and intervention outcomes in relation to our pre-specified progression criteria. All criteria but one were met to the green light-level, showing they were satisfactorily fulfilled. Only the monthly number of patients screened at stage 1 yielded possible concerns. This was defined as an issue that was likely to be present in the trial context only (problem Type A according to the ADePT terminology).

**Table 7:**
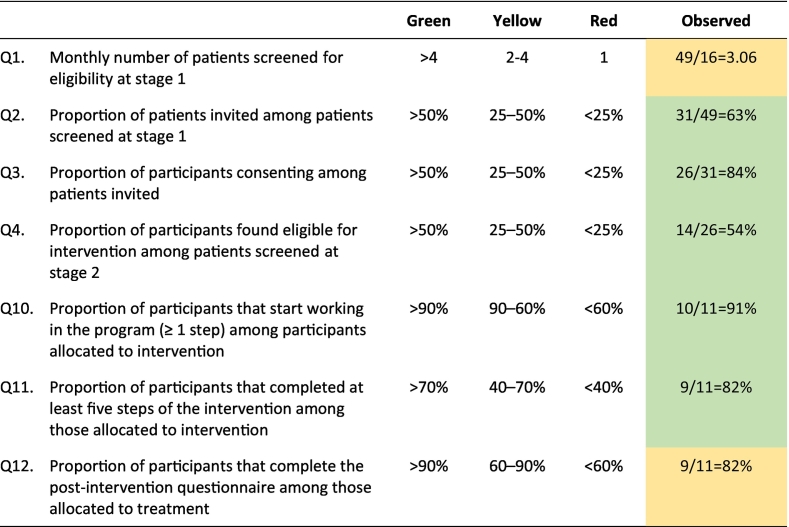
Pre-specified progression criteria of the current feasibility study.

Note. The progression criteria were set to be analysed separately rather than in conjunction.

## 4 Discussion

Overall, all but two progression criteria were fulfilled, signalling no concerns regarding feasibility and/or acceptance, and no need for further analysis or amendments. Two criteria, one relating to the feasibility of recruitment and one to the feasibility of intervention (intervention adherence) signalled possible concerns and need for further analysis, with a possible need for minor amendments (yellow light).

### 4.1 Feasibility of recruitment

All but two progression criteria were fulfilled (one of the relating to the feasibility of recruitment), implying that the proportion of patients invited, consenting and found eligible was acceptable. Only the number of patients screened at stage 1 per month, yielded possible concerns. Using the ADePT framework for analysis, this was found a problem mainly for the trial context. The research group identified inclusion of more study sites to be the solution with the greatest potential regarding its effectiveness and feasibility. To further increase the attractiveness of participation, we decided on using a design where all participants receive active treatment. Hence, the control group will be invited to the intervention with a delay of 10 weeks. In addition, we decided on minor amendments of the inclusion and exclusion criteria to be more inclusive. For example, patients with atrial fibrillation and severe lung- or kidney disease can be included in the RCT. On the other hand, patients with spontaneous coronary artery dissection, patients with a life expectancy of less than 1 year due to other severe conditions and expected poor compliance to behavioral therapy will be excluded.

### 4.2 Feasibility of data-collection

Participants needed a considerable time to finish the stage 2 screening, while pre- and post-test questionnaires were less time consuming. The risk of fatigue and reduced motivation for participation was discussed, but since the effort of completing questionnaires was not mentioned as a problem in interviews, the decision was to retain all screening questionnaires. Given that the intervention could be quite demanding as well, a screening that requires some effort could give a hint to the patients' capacity to adhere to the intervention.

Symptoms of stress and anxiety are generally known to decline over time post MI (Humphries et al., 2021). However, from screening to the pre-intervention assessment (2 weeks) the ratings on our most important outcomes were only marginally reduced and some even increased. However, from pre- to post intervention we noted a decline in symptom ratings with large effect sizes (*d* = 1.0–1.5) for our main outcomes (see Table 2). Seven out of 9 working in the program normalized their scores on one or both of the main outcomes. Although the un-controlled design makes it difficult to attribute symptom reduction to the intervention, our outcome measures seem sensitive enough to capture the changes in symptom load that might occur during the intervention period. It is likely that patients receiving only TAU also will improve during the months following their MINOCA, which has been taken into account in sample size calculations for the main trial.

### 4.3 Feasibility and experiences of the intervention

The results generally showed a high level of intervention adherence. Both quantitative and qualitative data indicated that participants worked actively and had positive experiences of the intervention program. In their systematic review of internet-delivered CBT interventions for patients with chronic illness, McCombie et al. (2015) suggest that both adherence and effect may benefit from disease specific interventions that are engaging and easy for the participant to relate to. Our intervention was designed in close collaboration with patient research partners and specifically tailored for this diagnostic group. Although one might argue that more generic interventions (e.g. one common intervention for all cardiac patients) might be easier to administer and implement, we believe that the high level of adherence seen in this feasibility study, in combination with the uniqueness of the MINOCA and TS diagnoses, support the idea of moving on to a full scale RCT trial evaluating this diagnosis specific intervention.

In this feasibility study, the only progression criteria relating to the feasibility of the intervention that was of potential concern was the proportion of participants that completed the post-intervention questionnaire among those allocated to treatment. While the figure of 82 % was lower than optimal, it was fond high enough not to raise strong concerns. With retention and adherence in mind, and in line with suggestions by McCombie et al. (2015), some reminders were used and were probably helpful. These were experienced as stressful by some, and helpful by others. As suggested by Knowles et al. (2014), both high and low levels of support can be experienced as either positive or negative. However, reminders and other forms of therapist support, both administrative and therapeutic, are usually helpful in internet based CBT, effecting both efficacy and treatment adherence (Andersson and Cuijpers, 2009; Baumeister et al., 2014; Johansson and Andersson, 2012; Richards and Richardson, 2012). Given that our participants asked for more personal support rather than less, we believe that the positive consequences of support are likely to outweigh negative ones, even though both viewpoints might co-occur in the participant group (Knowles et al., 2014). Taking into account the concern raised regarding the response rate at follow-up, and the previously identified importance of reminders in this regard (Andriopoulos et al., 2021), we have further reason to keep using reminders in the future RCT trial.

In the current study, reminders were manually elicited. This can be time consuming and might lead to reminders being missed. In the main study automatic reminders will be used, complemented by telephone calls to non-responders. However, therapist support will still be provided manually by clinical psychologists, supported by the recent finding that human guidance was more efficacious than technological guidance, both in terms of symptom reduction and adherence (Koelen et al., 2022).

Activating a new step after step 6 may have been experienced as unnecessarily demanding, as some participants never did. To reduce the risk of dropout, we thus decided to activate all intervention steps from start, giving participants the choice to proceed directly from step 6 to either step 7 or step 9. Some unclear parts of the texts were also brought to attention and will be revised accordingly. Since the personal contact with the psychologists was highly appreciated according to interviews, it was also decided to add another telephone call after step 3.

### 4.4 Limitations

The aim of this study was to address the feasibility of conducting an RCT and not to evaluate the effects of the intervention at hand. Hence, the study was neither powered nor designed to draw such inferences or generalize to other settings.

The COVID-19-pandemic affected recruitment negatively. Less patients were admitted to hospital for suspected MI in the first wave of the pandemic (De Rosa et al., 2020). Reasons for not seeking care were mostly fear of getting infected of COVID-19 at the hospital and concerns of not adding extra burden to the overcrowded hospitals (Lidin et al., 2021). The strain on hospital staff was also very high during an extensive time, which may also have had a negative effect on recruitment. The pandemic may also have influenced patient mood, willingness to participate and their way of using the intervention. Potentially it could also have had some positive effects, as digital tools were introduced and increasingly used in many areas of society during the pandemic. Which lead to a steep learning curve.

Offering an online intervention comes with many strengths. It provides access to services not otherwise available in-person, it can minimize the need for certain resources, and it enables flexibility for the participant to choose when and where they work through the program, overall delivering high rates of satisfaction and acceptability (Andrews et al., 2018). However, as expressed by one participant who had significant difficulties with concentration, digital formats can be perceived as being particularly demanding on maintaining focus and attention.

### 4.5 Strengths

This study was carried out as planned in spite of the pandemic. We managed to recruit participants that seemed to be relatively representative of the target group and that were similar to participants in previous studies. Since this study used the same procedures and context as the planned RCT, generalizability to the main trial is considered to be high.

Once participants had started working in the program, compliance was good. Nine out of 11 first allocated to intervention reached the predefined goal of completing five steps or more. Few were lost to follow-up. The combination of quantitative and qualitative research methods provided good insight to the issues of both feasibility and acceptance, and informed possible amendments to enhance these aspects further.

## 5 Conclusions

The study protocol was in general feasible with regard to recruitment, data collection and intervention. The project will now proceed to a full scale RCT, while making the following amendments to the protocol: include four additional study sites, increase the attractiveness by offering the intervention to the control group with a 10 week delay, revise exclusion criteria to be more inclusive, use automatic reminders, activate all intervention steps from start, revise unclear parts of the intervention material, and add one more telephone call from the psychologist.

## Funding

This project was funded by the 10.13039/501100004359Swedish Research Council [Grant 2018-02655]; The Swedish Heart Lung Foundation [Grant 20200221]; and the Swedish Heart and Lung Association [Grant 2019:41]. It was further sponsored by the 10.13039/501100004359Swedish Research Council also as part of the strategic research environment U-CARE [2009-1093].

## Declaration of competing interest

The authors declare that they have no known competing financial interests or personal relationships that could have appeared to influence the work reported in this paper.
